# Editorial: Hidden Secrets and Lessons From the Crystal Structures of Integral Membrane Proteins: Channels, Pumps and Receptors

**DOI:** 10.3389/fphys.2018.01466

**Published:** 2018-10-31

**Authors:** Mario Díaz, Garth L. Nicolson

**Affiliations:** ^1^Departamento de Biología Animal, Edafología y Geología, Facultad de Ciencias, Sección Biología, Universidad de La Laguna, Tenerife, Spain; ^2^Unidad Asociada de Investigación CSIC-Universidad de La Laguna “Fisiología y Biofísica de la Membrana Celular en Patologías Neurodegenerativas y Tumorales”, Tenerife, Spain; ^3^Institute for Molecular Medicine, Huntington Beach, CA, United States

**Keywords:** integral membrane proteins, crystal structure, structure dynamics, ion channels, active transporters, membrane exchangers, X-ray crystal and molecular structure

Our current understanding of the integral membrane protein structure and function has changed dramatically in the last two decades, thanks to novel experimental, and methodological approaches that take advantage of the latest technologies. From the earliest high-resolution crystallographic studies on bacteriorhodopsin (Deisenhofer et al., 1985) to newer studies on eukaryotic multi-subunit membrane proteins undergoing structural changes during their catalytic cycles, opening and closing, specificity, binding to modulators, and voltage-induced changes amongst other mechanistic events, membrane proteins have steadily started to reveal their longtime hidden structural secrets. This research topic intends to offer a wider view of our current understanding of the membrane protein structure and function that has emerged from the studies of crystal structures and other breakthrough methodologies, including focused reviews on the latest research in the field of membrane proteins, which includes protein crystallography, mass spectrometry, or cryo-electron microscopy (EM) and their usefulness to unravel important biological details of the structure-function relationships of membrane protein complexes, often leading to a better comprehension of their role in pathological events and human diseases.

## Methods

Membrane proteins represent a challenging family of macromolecules, particularly related to the methodology aimed at characterizing their three-dimensional structure. This is mostly due to their amphipathic nature as well as requirements of ligand bindings to stabilize or control their function. In this perspective article, Montenegro et al. argue on the usefulness of combining mass spectrometry and X-ray crystallography to unravel hidden mysteries of the membrane protein function. This technological implementation has helped to identify the overall stoichiometry of native-like membrane proteins complexed to ligand bindings as well as to provide insights into the transport mechanism across the membrane, as they illustrate several protein-lipid complexes (in particular transporters, ion channels, and molecular machines).

## Transporters and exchangers

Sun and Zheng focus their review on the unique properties of a dual function nitrate transporter NRT1.1, a member of the nitrate transporters superfamily in plants, which may switch between low- and high-affinity states to cope with the large nitrate fluctuation in soil. By means of structural studies on NRT1.1 in *Arabidopsis*, they show that single residue phosphorylation leads to changes in protein oligomerization. Notably, such secondary changes in protein oligomerization are sufficient to increase structural flexibility of NRT1.1 and to modify nitrate affinity. The authors propose a novel paradigm in which protein oligomerization and posttranslational modification can synergistically convey to modulate the functional capacity of dual-affinity transporters.

Recent structural and biophysical studies have shed light on the structural basis of ion transport and the allosteric regulation of sodium-calcium exchanger (NCX) proteins. The NCX proteins include a family of Na^+^/Ca^2+^ exchangers, which extrude Ca^2+^ from the cell to maintain cellular homeostasis. Within eukaryotic cells, NCX proteins exist as a complex set of orthologs, isoforms, and splice variants expressed in a tissue-specific manner. In this research topic, Giladi et al. review the structural basis for the diverse regulatory responses to Ca^2+^ binding in different orthologs and splice variants in relation to the dynamic of the two regulatory Ca^2+^-binding domains, CBD1 and CBD2.

Zhang et al. review the recent structural findings of proton-translocating nicotinamide nucleotide transhydrogenase (TH). Transhydrogenase is an enzyme complex in animal mitochondria and bacteria that utilizes the electrochemical proton gradient across membranes to drive the production of NADPH. The enzyme plays an important role in maintaining the redox balance of cells, and it has been shown to have implications in aging and human diseases. Structural insights into the mechanism of dynamics of TH require structural determinations of the protein complex in many conformational states. Here, Zhang et al. compile the information from recent technological breakthroughs in X-ray crystallography and cryo-EM to outline a comprehensive and detailed structural understanding of this very complex enzyme.

## Pumps

Clausen et al. review the various roles and expression patterns of the Na-K ATPase subunit (namely, the α, β, and FXYD subunits) isoforms. The Na-K ATPase was first described 60 years ago by Skou (1957), and it is perhaps the best primary active transporter studied so far. However, novel discoveries into the pump's atomic structure, cellular regulation, and pathophysiological roles continue to emerge. The distribution pattern of different isoforms is fine-tuned to cope with the different cellular needs, and mutations in the genes encoding for the different subunits/isoforms are associated with a plethora of pathophysiological effects, which are often linked to neurological diseases. Differences in the mutations of Na-K ATPase subunits (particularly in the α-subunit) affect kinetic parameters, pump regulation, and protein-protein/protein-lipid interactions.

## Ion channels

The major protein in the outer membrane of mitochondria is the voltage-dependent anion channel (VDAC), which mediates signal transmission across the outer membrane but also the exchange of metabolites, most importantly ADP and ATP. Over the past 10 years, complementary structural and functional information on proteins of the VDAC superfamily have been collected from *in organello, in vitro*, and *in silico* studies. Most of these findings have confirmed the validity of the original structures (19-stranded anti-parallel beta-barrel with an N-terminal helix located inside). The article by Zeth and Zachariae reviews the most important advances on the structure and function of VDAC superfamily members, and the review summarizes how they enhanced our understanding of the channel.

The mammalian Sec61 complex existing in the membrane of the endoplasmic reticulum (ER) forms a dynamic gated channel, which provides an aqueous path for nascent polypeptides in the cytosol into the ER lumen and is regulated by various allosteric effectors. Further, when the pore forming subunit of the complex (gated pathway) is open, it also provides a pathway for the efflux of calcium ions from the ER into the cytosol. Lang et al. suggest that this feature is linked to the regulation of ATP import into the ER and the initiation of the intrinsic pathway to apoptosis. Recently, cryoelectron tomography of translocons in native ER membrane vesicles has given unprecedented insights into the architecture and dynamics of the native translocon harboring the Sec61 channel. In this review by Lang et al., this structural information is discussed in light of different Sec61 channel activities including ribosome receptor function, membrane insertion, and translocation of newly synthesized polypeptides as well as the putative physiological roles of the Sec61 channel as a passive ER calcium leak channel.

A total of 27 renowned researchers from seven countries (Denmark, United Kingdom, USA, Chile, Netherlands, Germany, and Israel) have contributed to this research topic. We hope this compilation provides a stimulating knowledge base for upcoming research in this very active and promising investigation field.

## Author contributions

MD and GN have edited the research topic and have written and drafted the manuscript.

### Conflict of interest statement

The authors declare that the research was conducted in the absence of any commercial or financial relationships that could be construed as a potential conflict of interest.
